# Radiomics analysis of gadoxetic acid-enhanced MRI for evaluating vessels encapsulating tumour clusters in hepatocellular carcinoma

**DOI:** 10.3389/fonc.2024.1422119

**Published:** 2024-08-13

**Authors:** Jiyun Zhang, Maotong Liu, Qi Qu, Mengtian Lu, Zixin Liu, Zuyi Yan, Lei Xu, Chunyan Gu, Xueqin Zhang, Tao Zhang

**Affiliations:** ^1^ Department of Radiology, Affiliated Nantong Hospital 3 of Nantong University, Nantong Third People’s Hospital, Nantong, Jiangsu, China; ^2^ Department of Pathology, Affiliated Nantong Hospital 3 of Nantong University, Nantong Third People’s Hospital, Nantong, Jiangsu, China

**Keywords:** hepatocellular carcinoma, vessels encapsulating tumour clusters, radiomics, gadoxetic acid, magnetic resonance imaging

## Abstract

**Purpose:**

The aim of this study was to develop an integrated model that combines clinical-radiologic and radiomics features based on gadoxetic acid-enhanced MRI for preoperative evaluating of vessels encapsulating tumour clusters (VETC) patterns in hepatocellular carcinoma (HCC).

**Methods:**

This retrospective study encompassed 234 patients who underwent surgical resection. Among them, 101 patients exhibited VETC-positive HCC, while 133 patients displayed VETC-negative HCC. Volumes of interest were manually delineated for entire tumour regions in the arterial phase (AP), portal phase (PP), and hepatobiliary phase (HBP) images. Independent predictors for VETC were identified through least absolute shrinkage and selection operator (LASSO) regression and multivariable logistic regression analysis, utilising radiomics-AP, PP, HBP, along with 24 imaging features and 19 clinical characteristics. Subsequently, the clinico-radiologic model, radiomics model, and integrated model were established, with a nomogram visualising the integrated model. The performance for VETC prediction was evaluated using a receiver operating characteristic curve.

**Results:**

The integrated model, composed of 3 selected traditional imaging features (necrosis or severe ischemia [OR=2.457], peripheral washout [OR=1.678], LLR_AP (Lesion to liver ratio_AP) [OR=0.433] and radiomics-AP [OR=2.870], radiomics-HBP [OR=2.023], radiomics-PP [OR=1.546]), showcased good accuracy in predicting VETC patterns in both the training (AUC=0.873, 95% confidence interval [CI]: 0.821-0.925)) and validation (AUC=0.869, 95% CI:0.789-0.950) cohorts.

**Conclusion:**

This study established an integrated model that combines traditional imaging features and radiomic features from gadoxetic acid-enhanced MRI, demonstrating good performance in predicting VETC patterns.

## 1 Introduction

Hepatocellular carcinoma (HCC) stands as one of the most prevalent primary hepatic malignancies, with an annual global incidence of approximately 500,000 cases, a figure that continues to ascend each year (1, 2). Surgical resection and liver transplantation offer potential curative options for HCC patients, however such patients have high recurrence rates (3), and the precise underlying mechanisms governing metastasis and recurrence remain incompletely elucidated.

Epithelial-mesenchymal transition (EMT), a classical metastatic mechanism, is intricately linked to cancer recurrence and metastasis, and has been correlated with shortened survival among HCC patients (4). EMT-mediated metastasis has been identified as a driver of microvascular invasion (MVI), a crucial prognostic factor for HCC post-surgical intervention (5). Nevertheless, within clinical realms, a notable proportion of patients, devoid of MVI and other high-risk conventional clinical parameters, experience relapses subsequent to therapeutic resection. This underscores the inadequacy in comprehensively depicting the heterogeneity and underpinning mechanisms of HCC metastasis and recurrence.

Fang et al. (6) present an alternative mode of HCC metastasis, termed as the EMT-independent “vessels encapsulating tumour clusters” (VETC), characterised by a sinusoidal network of functional blood vessels that entirely envelop sections of the primary tumour. The VETC phenotype, deemed a dependable pathological parameter, exerts an impact on survival and exhibits significant associations with overall survival (OS) and early recurrence (6–9). The comprehension of HCC’s VETC status could indeed have far-reaching implications for future treatments (10), to be able to apply precision medicine to HCC treatment.

Fang et al. (11)have indicated that the VETC pattern might serve as a dependable marker for selecting HCC patients who could potentially benefit from sorafenib treatment. VETC-positive tumours might respond to lenvatinib treatment because of gene expression analyses of VETC-positive tumours (10, 12, 13). Because VETC formation is dependent on Ang2, Ang2 inhibitors may be promising in the treatment of VETC-positive HCC. VETC-positive tumours are associated with CTNNB1 mutations and the Wnt/b-catenin signalling pathway activation (7, 12–14). Lin et al. (15) demonstrated significant benefits of adjuvant TACE in terms of time to recurrence and OS in VETC-positive HCC patients. Therefore, VETC status may provide guidance for the prevention and treatment of HCC recurrence. However, the postoperative diagnosis of VETC necessitates surgical specimens. Consequently, it holds immense importance to develop the ability to preoperatively evaluate the VETC pattern, necessitating further research.

To the best of our knowledge, only a handful of studies have endeavoured to delineate the imaging characteristics of the VETC pattern in HCC (9, 16–18). Prior investigations (9, 16–18) have showcased that preoperative CT or MRI features could be harnessed to characterise the VETC pattern. However, despite its potential utility, the analysis of image features is inherently subjective and suffers from limitations in terms of repeatability. Radiomics, a burgeoning field of imaging analysis, can extract a multitude of high-dimensional quantitative features from multimodal medical images, subsequently unravelling the connections between these features and the tumour’s diagnosis, pathology, and prognosis (19). Recently, a limited number of studies have employed radiomics to forecast the VETC pattern based on MRI (20–23). Yu et al. (20) formulated a gadoxetic acid-enhanced MRI radiomics model to preoperatively predict VETC, 1,316 quantitative features from intratumoural and peritumoural regions of HCCs were extracted and used four different machine learning algorithms to generate impressive AUROCs (>0.90). Chu et al. (22), on the other hand, employed a deep learning framework based on 3D CNN for multitask learning to predict VETC using preoperative gadoxetic acid-enhanced MRI, albeit with a focus on HBP phases that did not encompass multiphase contrast-enhanced MRI. Recently, Xue Dong et al (23) employed dynamic contrast-enhanced MRI with extracellular contrast agents to construct models, demonstrating that a deep learning radiomic model based on the peritumoural portal phase yielded optimal performance. However, there is no widely accepted criteria for preoperative imaging diagnosis of VETC, and more studies are needed to find more robust evidence.

In this investigation, we conducted an analysis of MR images following the Liver Imaging Reporting and Data System (LI-RADS) v2018 (24) guidelines based on gadoxetic acid–enhanced MRI. Subsequently, these findings were amalgamated with radiomics data to formulate predictions regarding the VETC pattern and overall patient prognosis in cases of HCC.

Hence, the principal aim of this research was to formulate an integrated model that fuses clinical-radiologic data and radiomics-derived features on gadoxetic acid-enhanced MRI scans. This model serves the purpose of preoperatively evaluating the VETC pattern in hepatocellular carcinoma (HCC).

## 2 Materials and methods

### 2.1 Patients

**Figure 1:**
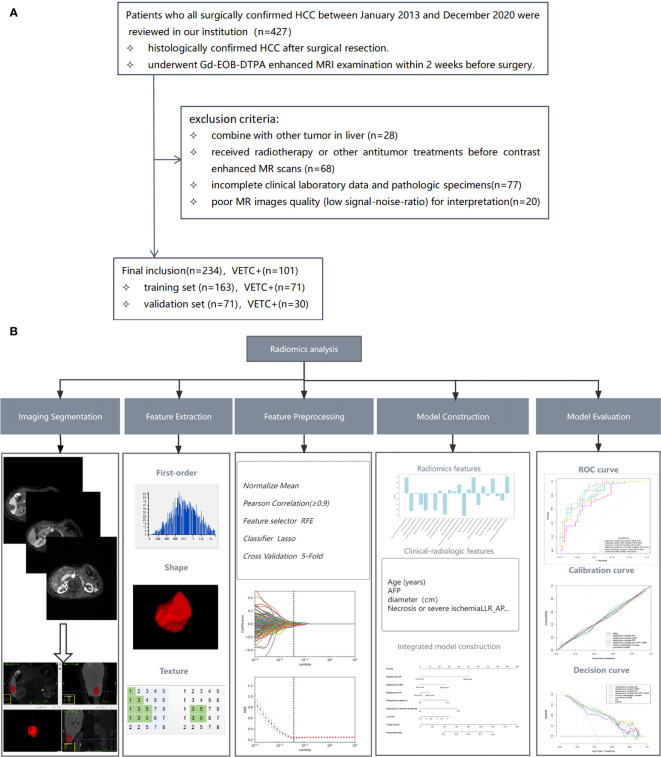
Flowchart illustrating the patient selection process **(A)** and the workflow for radiomics analysis **(B)**. *RFE*, recursive feature elimination; *LASSO*, least absolute shrinkage and selection operator; *AFP*, alpha-fetoprotein; *LLR*, lesion to liver ratio; *AP*, arterial phase; *ROC*, receiver operating characteristic.

This retrospective study was conducted at a sole medical institution. Ethical clearance was obtained from the local institutional ethics review board, written informed consent was waived. Our investigation focused on patients who underwent surgical confirmation of HCC between January 2013 and December 2020 within our institution. Detailed criteria for inclusion and exclusion are provided in **Figure 1A**. Enrolled patients were randomly partitioned into a training set and a validation set in a 7:3 ratio, as illustrated in **Figure 1A**.

### 2.2 Clinical characteristics

Clinical variables and preoperative laboratory data were meticulously extracted from the patients’ medical records, as outlined in **Table 1**. All clinical and laboratory data were procured within a one-week window prior to or subsequent to the gadoxetic acid-enhanced MRI examination.

**Table 1:** Baseline clinical characteristics and pathological parameters and radiologic factors of the training and validation set.

Characteristics	Training set			Validation set		
	VETC-(92)	VETC+(71)	P -value *	VETC-(41)	VETC+(30)	P- value *
Age (years) *b*	60 (52, 67)	57 (51, 64)	0.072	62.00 (53.00, 65.00)	54.00 (51.00, 59.00)	0.033
Sex (male)	64 (69.57)	46 (64.79)	0.519	29 (70.73)	27 (90)	0.05
AFP *b*	8.18 (3.64, 59.94)	19.00 (3.18, 300.00)	0.201	8.82 (4.53, 101.10)	17.65 (3.56, 97.24)	0.839
PIVKA-II *b*	44.36 (21.47, 179.42)	162.67 (61.45, 384.36)	<0.001	61.07 (25.34, 184.32)	88.28 (40.37, 502.90)	0.068
ALB *a*	40.20 ± 5.02	40.24 ± 4.32	0.955	41.84 ± 5.84	40.84 ± 4.83	0.445
TBIL *b*	17.00 (12.45, 21.08)	15.30 (11.30, 20.20)	0.241	16.30 (12.70, 19.60)	18.30 (13.10, 22.40)	0.264
ALT *b*	30.50 (22.00, 56.50)	35.00 (20.00, 48.00)	0.749	29.00 (22.00, 43.00)	36.00 (22.00, 67.00)	0.511
AST *b*	37.00 (26.00, 56.00)	37.00 (25.00, 51.00)	0.679	37.00 (28.00, 48.00)	36.50 (25.00, 57.00)	0.903
ALP *b*	84.00 (65.00, 108.00)	94.00 (60.00, 112.00)	0.861	93.00 (72.50, 123.00)	80.50 (66.00, 110.00)	0.116
GGT *b*	40.50 (27.00, 72.00)	45.00 (31.00, 70.00)	0.422	56.00 (30.00, 96.00)	37.00 (25.00, 96.00)	0.744
PLT *b*	112.50 (74.50, 158.50)	119.00 (86.00, 173.00)	0.549	117.00 (78.00, 151.00)	100.00 (71.00, 145.00)	0.284
PT *b*	11.85 (10.95, 13.30)	12.00 (11.40, 12.60)	0.489	11.90 (11.10, 12.90)	11.95 (11.10, 12.80)	0.784
APRI *b*	0.39 (0.19, 0.69)	0.30 (0.21, 0.51)	0.429	0.34 (0.20, 0.66)	0.38 (0.25, 0.58)	0.629
Cause of liver disease (HBV,others)	85, 7	65,6	0.844	35,6	26,4	1
Child-Pugh (A,B)	81,11	61,10	0.688	36,5	22,8	0.119
Pathological						
Diameter (cm)b	2.45 (1.40, 3.90)	3.00 (2.00, 4.50)	0.017	2.70 (1.60, 4.00)	2.70 (2.00, 5.80)	0.5642
Number of tumour (single,multi)	79,13	58,13	0.47	36,5	19,11	0.015
MVI (%)	82 (89.13)	41 (57.74)	<0.001	33 (80.49)	15 (50)	0.007
Edmondson grade (I-II,III-IV)	31,61	5,66	<0.001	8,33	2,28	0.174
MTM (%)	0	10 (14.08)	<0.001	0	2 (6.67)	0.175
Capsule infiltration	4 (4.35)	9 (12.68)	0.052	3 (7.32)	5 (16.67)	0.269
Inflammatory infiltrates	75 (81.52)	44 (61.97)	0.005	31 (75.61)	19 (63.33)	0.263
Gross vascular invasion	3 (3.26)	3 (4.23)	1	12.44)	2 (6.67)	0.57
Satellite nodule	1 (1.09)	2 (2.82)	0.581	2 (4.88)	4 (13.33)	0.233
CK7+ (%)	51 (55.43)	32 (45.07)	0.189	27 (65.85)	16 (53.33)	0.286
CK19+ (%)	24 (26.09)	16 (22.54)	0.601	13 (31.71)	7 (23.33)	0.438
Radiology Factors						
LI-RADS major features						
Nonrim arterial phase hyperenhancement (%)	60 (65.22)	36 (50.70)	0.062	23 (56.10)	14 (46.67)	0.432
Nonperipheral washout (%)	61 (66.30)	36 (50.70)	0.044	23 (56.10)	14 (46.67)	0.432
Enhancing capsule (%)	58 (63.04)	55 (77.46)	0.048	35 (85.37)	27 (90)	0.724
LI-RADS ancillary features (favouring HCC in particular)						
Nonenhancing capsule (%)	3 (3.26)	5 (7.04)	0.297	0 (0.00)	2 (6.57)	0.175
Mosaic architecture (%)	10 (10.87)	6 (8.45)	0.607	5 (12.20)	2 (6.57)	0.691
Nodule-in-nodule architecture (%)	7 (7.61)	1 (1.41)	0.139	3 (7.32)	0 (0.00)	0.258
Fat in mass (%)	14 (15.22)	16 (22.54)	0.232	8 (19.51)	6 (20)	0.959
Blood products in mass (%)	12 (13.04)	12 (16.90)	0.491	8 (19.51)	6 (20)	0.959
LI-RADS ancillary features (favouring malignancy, not HCC in particular)						
Transitional phase hypointensity (%)	91 (98.91)	70 (98.59)	1	40 (97.56)	30 (100)	1
Restricted diffusion (%)	91 (98.91)	71 (100)	1	41 (100)	30 (100)	1
Mild-moderate T2 hyperintensity (%)	91 (98.91)	71 (100)	1	41 (100)	30 (100)	1
Corona enhancement (%)	23 (25)	25 (35.21)	0.156	16 (39.02)	9 (30)	0.432
Fat sparing in solid mass (%)	77 (83.70)	52 (73.24)	0.103	33 (80.49)	24 (80)	0.959
Hepatobiliary phase hypointensity (%)	92 (100)	71 (100)	1	40 (97.56)	30 (100)	1
Iron sparing in solid mass (%)	88 (95.65)	70 (98.59)	0.388	41 (100)	29 (96.67)	0.423
LI-RADS M features						
Rim arterial phase hyperenhancement (%)	28 (30.43)	35 (49.30)	0.014	17 (41.46)	14 (46.67)	0.662
Portal phase peripheral washout (%)	10 (10.87)	30 (42.25)	0	10 (24.39)	9 (30)	0.598
Delayed central enhancement (%)	6 (6.52)	2 (2.82)	0.468	0 (0.00)	0 (0.00)	0.598
Targetoid TP or HBP appearance (%)	20 (21.74)	14 (19.72)	0.753	12 (29.27)	6 (20)	0.375
Targetoid restriction (%)	21 (22.83)	21 (29.58)	0.329	13 (31.71)	9 (30)	0.878
Necrosis or severe ischemia (%)	15 (16.30)	31 (43.66)	<0.001	7 (17.07)	13 (43.33)	0.015
Infiltrative appearance (%)	14 (15.22)	13 (18.31)	0.599	6 (14.63)	4 (13.33)	1
Non LI-RADS high-risk features						
Non-Smooth tumour margin (%)	20 (21.74)	22 (30.99)	0.181	12 (69.27)	7 (23.33)	0.577
Peritumoural hypointensity on HBP (%)	24 (26.09)	27 (38.03)	0.103	19 (46.34)	13 (43.33)	0.801
Quantitative indicators						
LLR_AP *b*	1.37 (1.21, 1.58)	1.17 (1.09, 1.36)	0.014	1.50 (1.23, 1.73)	1.12 (1.03, 1.24)	<0.001
LLR_PP *b*	0.55 (0.46, 0.62)	0.53 (0.46, 0.63)	0.398	0.56 (0.48, 0.62)	0.48 (0.46, 0.59)	0.107
LLR_HBP *b*	0.89 (0.67, 1.09)	0.78 (0.65, 1.09)	0.044	0.98 (0.79, 1.26)	0.75 (0.69, 0.97)	<0.001

Categorical variables are presented as N (%) according to different levels.

a Data are presented as mean ± standard deviation.

b Data are presented as median (interquartile range).

VETC, vessels encapsulating tumour clusters; AFP, alpha-fetoprotein; PIVKA-II, protein induced by vitamin K absence-II; ALT, alanine aminotransferase; AST, aspartate aminotransferase; ALB, serum albumin; TB, serum total bilirubin; GGT, gamma-glutamyl transferase; ALP, alkaline phosphatase; PLT, platelet count; PT, prothrombin time; APRI=AST/PLT; HBV, hepatitis B virus; MVI, microvascular invasion; MTM, macrotrabecular-massive; LI-RADS, Liver Imaging Reporting and Data System; LLR, lesion to liver ratio; AP, arterial phase; PP, portal phase; HBP, hepatobiliary phase; TP, transitional phase.

^*^ T test results for continuous variables with normal distribution, Wilcoxon test results for continuous variables with abnormal distribution, and chisquare test or Fisher exact test results for categorical variables.

### 2.3 Histopathology

All surgical specimens underwent thorough evaluation by an accomplished pathologist (G.C.Y.) with over two decades of experience in the analysis of hepatic pathology. The VETC pattern is characterised by the presence of sinusoid-like vessels that form intricate network patterns, encapsulating individual clusters of tumour cells, either in their entirety or partially within the tumour. This pattern was discerned through CD34 immunostaining during imaging (25). In this study, tumour sections displaying a discernible VETC pattern, accounting for a minimum of 5% of the tumour’s area, were classified as VETC+. Additionally, the other histopathological attributes were documented in **Table 1**.

### 2.4 Follow up

Post-surgery, patients were subjected to regular follow-up appointments spaced between intervals of 3 to 6 months. These sessions involved assessments based on alpha-fetoprotein (AFP) levels, ultrasonography, and either CT or MRI. Recurrence was determined by the appearance of new intrahepatic lesions and/or instances of extrahepatic metastases. The culmination of the follow-up period was in April 2022. The duration between the surgical intervention date and the earliest manifestation of tumour recurrence defined the recurrence-free survival (RFS) period.

### 2.5 Magnetic resonance imaging examination

Detailed information regarding the MRI equipment and parameters can be found in **Supplementary Text 1** and **Supplementary Table 1**.

### 2.6 Analysis of radiologic features

Two radiologists, referred to as Reader 1 (M.T.L.) and Reader 2 (L.X.) endowed with professional experience of 6 years and 10 years respectively, independently scrutinised all imaging data and gauged the corresponding parameters, blinded to clinical, laboratory, and pathologic information. Following the individual assessments, the two readers convened two weeks later for a collective review. Any disparities that emerged were harmonised through intervention by a senior radiologist (X.Q.Z.), who possessed 18-year experience in abdominal MRI diagnostics. While individual scores were utilised for the computation of inter-observer concordance, consensus scores were employed for the ultimate analysis.

The imaging attributes encompassed both major and ancillary features, consistent with the LI-RADS v2018 (24). Moreover, a range of other high-risk imaging features of interest (as outlined in **Table 1**) were included. A detailed exposition of the imaging feature definitions can be found in **Supplementary Table 2**. When patients have multiple lesions, the largest substantial lesion was selected for analyses.

Quantitative assessment was employed to evaluate the signal intensity (SI) of lesion-to-liver ratio (LLR), expressed as LLR = SI_tumor_/SI_liver_. Additional particulars are elaborated upon in **Supplementary Text 2**.

### 2.7 Tumour segmentation and radiomics feature extraction

The radiomic analysis process is illustrated in **Figure 1B**. Gd-EOB-DTPA-enhanced MR AP, PP and HBP images were exported as digital imaging data and communications in medicine (DICOM) for mat. All images were transferred into the ITK-SNAP software (www.itksnap.org) for tumour segmentation. A team of two radiologists, known as Reader 3 (Q.Q.) and Reader 4 (J.Y.Z.), each with professional experience spanning 4 years and 6 years respectively, undertook the task of manually delineating three- dimensional volumes of interest (VOI) for the entire tumour. The ROI was manually delineated on each axial slice of the AP, PP, and HBP images, covering the entire tumour, and finally a volume of interest (VOI) representing the tumour area was presented. To ensure robustness, 30 patients were randomly selected and independently reviewed by both Reader 3 and Reader 4, from which interobserver intra-class correlation coefficients (ICC) were computed,ICC≥0.8 indicated high consistency, 0.5–0.79 middle, and<0.5 low. Moreover, within this subset, Reader 4 repeated the process of tumour segmentation and intraobserver ICCs were calculated. The subsequent segmentation of the remaining images was exclusively performed by Reader 4.

Utilising an open-source software package (https://github.com/salan668/FAE), all radiomics features were extracted and preprocessed. For each MRI sequence (including arterial phase (AP), portal phase (PP), and hepatobiliary phase (HBP) imaging), a total of 1050 quantitative radiomics features were extracted from the VOI of the tumour. These encompassed first-order statistics, shape, and texture features. Texture features comprised gray level co-occurrence matrix (GLCM), gray level run length matrix (GLRLM), gray level size zone matrix (GLSZM), gray level dependence matrix (GLDM), and neighboring gray tone difference matrix (NGTDM). Features exhibiting ICC values greater than 0.80 were selected for subsequent analysis. A Pearson correlation coefficients (PCCs) test was subsequently executed, and features boasting coefficients exceeding 0.90 were excluded to mitigate redundancy. Then we used the least absolute shrinkage and selection operator (LASSO) to reduce the redundancy and dimensionality of the features of each single sequence and to determine the hyper-parameter (e.g. the number of features) of model, we applied cross validation with 5-fold on the training data set. Under the optimal lambda value, features exhibiting non-zero characteristic coefficients were identified and subsequently utilised. These features denoted the correlation between the radiomic feature and VETC pattern, contributing to the final establishment of the radiomics model.

### 2.8 Model construction and validation

For radiomics model, we analysed each single sequence to built model separately and combine model, namely radiomics model_AP, radiomics model_PP, radiomics model_HBP, and radiomics model_AP+PP+HBP. The threshold of radiomics model was determined using receiver operating characteristic (ROC) analysis by maximising the Youden index.

Clinical characteristics and radiologic features with a *P* value <0.05 in univariate logistic regression analysis were entered into multivariate logistic regression analysis using the stepwise method. Akaike’s information criterion (AIC) was used for model reduction, and the model with the minimum AIC value was used to generate the clinico-radiologic model.

Subsequently, the radiomics features and clinico-radiologic features were amalgamated to establish the integrated model, visualised through a nomogram. The nomogram was built based on each *β* regression coefficient in the multivariable regression model. Model performance was assessed via the receiver operating characteristic (ROC) curve. The optimal threshold was identified through ROC analysis, aiming to maximise the Youden index. The area under the curve (AUC), accuracy, sensitivity, specificity, positive predictive value (PPV), and negative predictive value (NPV) were computed for evaluation. Calibration curves were employed to assess the performance of the nomogram. Furthermore, the clinical utility of the nomograms was evaluated by quantifying the net benefits through decision curve analysis (DCA).

### 2.9 Statistical analysis

Continuous variables were expressed as means with corresponding standard deviations or medians with interquartile ranges, while categorical data were represented as numbers and percentages. The Student’s t-test or Mann-Whitney U-test was utilised for comparing continuous variables, while Fisher’s exact test or χ^2^ test was employed for categorical variables. The Cohen’s kappa statistic was employed to assess interobserver agreement for traditional radiologic features. The interpretation of kappa values followed the criteria: κ > 0.80 denoting excellent agreement, 0.50 ≤ κ ≤ 0.80 representing good agreement, and κ < 0.50 indicating poor agreement.

The Kaplan-Meier method was utilised to compute RFS and generate survival curves, with the log-rank test used for assessing statistical significance. All statistical analyses were conducted utilising R software (version 3.6.0) and SPSS 20.0. A significance level of <0.05 was adopted for all analyses.

## 3 Results

### 3.1 Basic clinico-radiologic characteristics

A total of 234 cases were encompassed within this study, of which 101 (43.16%) were pathologically ascribed to possess the VETC pattern. Comprehensive clinical characteristics pertaining to both the training and testing cohorts are outlined in **Table 1**. The majority of clinical characteristics and radiological factors exhibited no statistically significant disparities between the training and validation sets, as delineated in **Supplementary Table 3**.

Several clinical attributes displayed notable discrepancies between the VETC-positive and VETC-negative groups (**Supplementary Table 3**). The ICC stood at ≥0.8 for 62.96% of radiological features, and within the range of 0.5–0.79 for 37.04% of features (**Supplementary Table 4**).

### 3.2 Development of VETC-predicting models

#### 3.2.1 Clinico-radiologic model

The multivariable logistic regression unveiled peripheral washout (OR = 6.493; 95% CI: 2.485-16.967), necrosis or severe ischemia (OR = 4.756; 95% CI: 1.964-11.516), targetoid TP or HBP appearance (OR = 1.307; 95% CI: 0.101-1.935), and LLR_AP (OR = 0.082; 95% CI: 0.012-0.459) as independent risk factors for the VETC pattern in **Supplementary Table 5**.

#### 3.2.2 Radiomics model

Initially, 966 AP radiomics features, 924 PP radiomics features, and 987 HBP radiomics features were included, each displaying ICC exceeding 0.8. Ultimately, 8 AP radiomics features, 3 PP radiomics features, and 11 HBP radiomics features were identified, as illustrated in **Supplementary Figure 1**. The resultant radiomics model (R-score) is expounded upon in **Supplementary Table 6**.

#### 3.2.3 Integrated model

**Figure 2:**
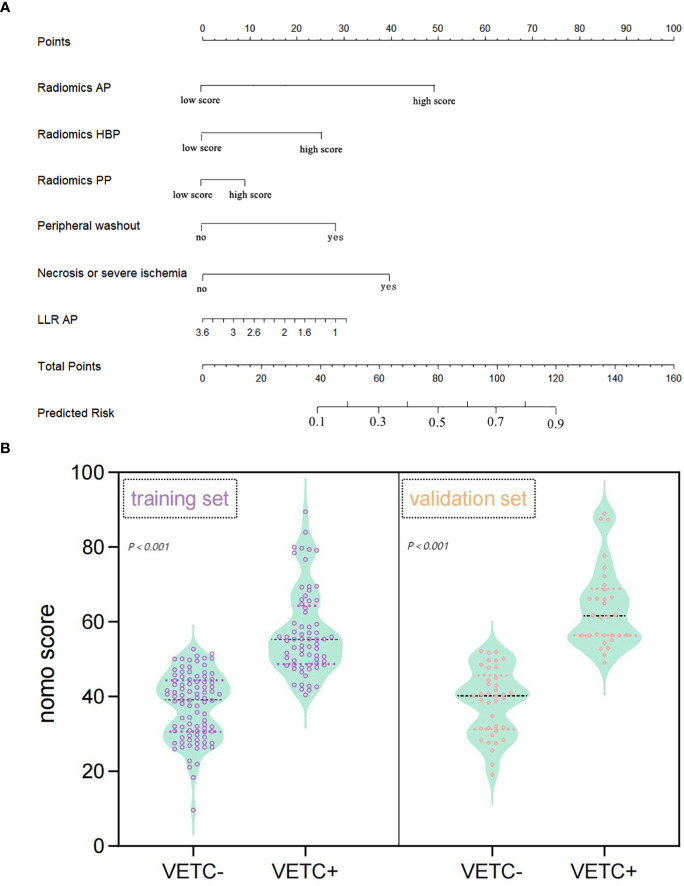
Nomogram representing the integrated model for evaluating the VETC pattern, alongside a violin plot depicting nomo-score distribution in VETC+ and VETC- groups. **(A)** The nomogram displays proportional regression coefficients of individual predictors. **(B)** Wilcoxon test demonstrates significant distinction in Nomo scores between VETC+ and VETC- groups based on the integrated model, observed in both the training and validation sets (*p*<0.01). *VETC*, vessels encapsulating tumour clusters; *LLR*, lesion to liver ratio; *AP*, arterial phase; *PP*, portal phase; *HBP*, hepatobiliary phase.

In the multivariate regression analysis, radiomics-AP (OR = 2.87; 95% CI: 1.293-3.704), radiomics-HBP (OR = 2.023; 95% CI: 1.012-3.398), radiomics-PP (OR = 1.546; 95% CI: 0.089-1.882), necrosis or severe ischemia (OR = 2.457; 95% CI: 1.133-7.367), peripheral washout (OR = 1.678; 95% CI: 1.046-2.423), and LLR_AP (OR = 0.433; 95% CI: 0.221-0.435) emerged as independent prognostic factors for histologic VETC (**Table 2**, **Figure 2A**). Notably, the Wilcoxon test exhibited a significant distinction between the Nomo scores of VETC+ and VETC- classifications, as determined by the integrated model (p < 0.01) (**Figure 2B**).

**Table 2:** Univariate and multivariate analysis of preoperative radiomics and clinico-radiologic factors in prediction VETC.

Factors	Univariate analysis		Multivariate analysis	
	*p* value	OR (95% CI)	*p* value	OR (95% CI)
Radiomics-AP	<0.001	4.074 (1.661-2.59)	<0.001	2.870 (1.293-3.704)
Radiomics-PP	<0.001	0.672 (0.053-0.962)	0.047	1.546 (0.089-1.882)
Radiomics-HBP	<0.001	1.900 (0.266-2.703)	0.025	2.023 (1.012-3.398)
Diameter cm	0.027	1.203 (1.021-1.416)		
Peripheral washout	<0.001	4.738 (2.146-10.459)	0.034	1.678 (1.046-2.423)
Targetoid TP or HBP appearance	0.037	2.365 (1.053-5.311)		
Necrosis or severe ischemia	<0.001	4.833 (2.238-10.441)	<0.001	2.457 (1.133-7.367)
LLR_AP	<0.001	0.026 (0.004-0.148)	0.021	0.433 (0.221-0.435)

VETC, Vessels encapsulating tumour clusters; LLR, Lesion to liver ratio; AP, arterial phase; PP, portal phase; HBP, hepatobiliary phase; TP, transitional phase; CI, Confidence interval; OR, Odds ratio.

### 3.3 Performance of the three models in the training and validation set

**Figure 3:**
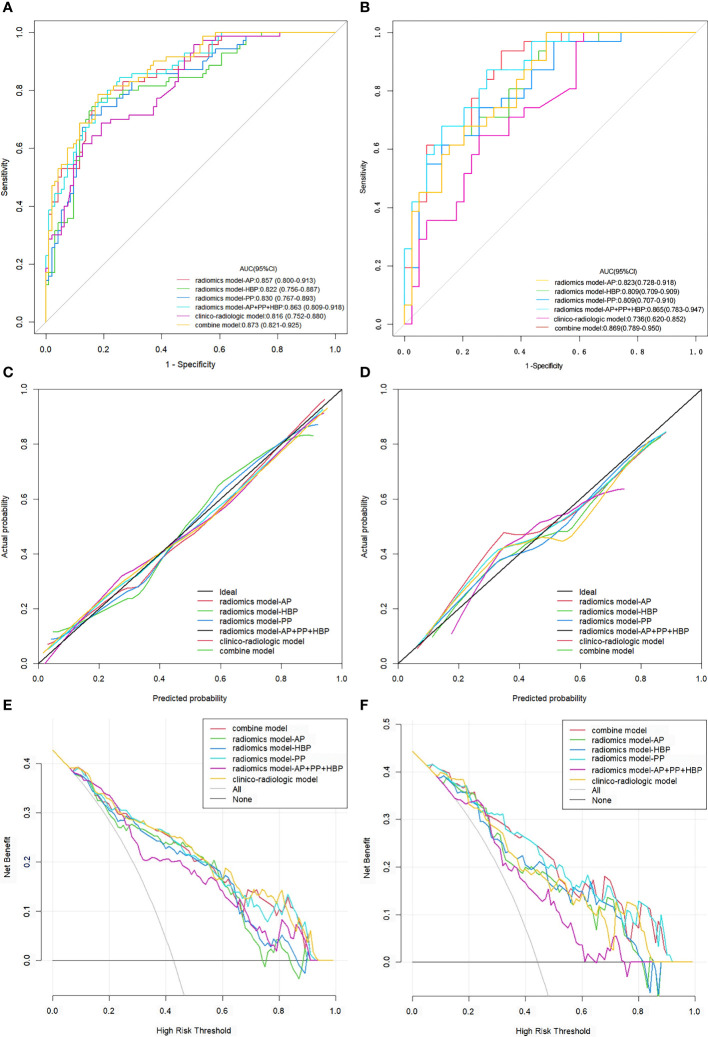
ROC curves, calibration curves, and DCA for various models evaluating the VETC pattern. **(A, B)** ROC curves for VETC pattern assessment in the training set **(A)** and validation set **(B)**. **(C, D)** Calibration curves for VETC assessment in the training **(C)** and validation set **(D)**.The diagonal 45-degree line indicates perfect prediction. **(E, F)** DCA for VETC assessment in the training **(E)** and validation set **(F)**. The vertical axis represents the value of net benefit, and the horizontal axis represents the threshold level. The coloured line is the expected net benefit of per patient based on each models. *ROC*, receiver operating characteristics; *DCA*, decision curve analysis; *VETC*, vessels encapsulating tumour clusters; *AUC*, area under the curve; *AP*, arterial phase; *PP*, portal phase; *HBP*, hepatobiliary phase.

**Table 3** succinctly presents the predictive performance metrics of the clinico-radiologic model, radiomics model, integrated model, and individual risk factors. The AUC of the clinical-radiologic model was lower than that of the radiomics model and integrated model (Z = 2.501 and 3.063, *p* = 0.012 and 0.002) in the training cohort and (Z = 2.497 and 3.200, *p* = 0.013 and 0.001) in the validation cohort, while the AUC of the radiomics model was inferior to the integrated model (Z = 0.422, *p* = 0.673) in the training cohort and (Z = 0.164, *p* = 0.870) in the validation cohort (**Figures 3A, B**).

**Table 3:** Performance of the three models and risk factors in the training and validation sets.

	Cutoff*	Training set				Validation set			
Models		AUC(95% CI)	Sensitivity	Specificity	Accuracy	AUC(95% CI)	Sensitivity	Specificity	Accuracy
Peripheral.washout	_	0.734 (0.568-0.700)	38.57% (28.03-50.30)	88.30% (80.09-93.50)	67.07% (59.55-73.81)	0.707 (0.498-0.716)	41.94% (26.39-59.26)	79.49% (64.21-89.47)	62.86% (51.13-73.25)
Necrosis or severe ischemia	_	0.743 (0.576-0.711)	41.43% (30.62-53.13)	87.23% (78.85-92.69)	67.68% (60.18-74.38)	0.714 (0.501-0.727)	48.39% (31.97-65.16)	74.36% (58.76-85.59)	62.86% (51.13-73.25)
LLR_AP	1.123	0.671 (0.587-0.755)	95.71% (87.65-99.02)	51.06% (41.12-60.93)	70.12% (62.71-76.62)	0.675 (0.546-0.804)	100.00% (86.91-100.00)	48.72% (33.86-63.80)	71.43% (59.89-80.73)
Clinico-radiologic model	0.464	0.816 (0.752-0.880)	68.57% (56.93-78.28)	80.85% (71.66-87.62)	75.61% (68.47-81.57)	0.736 (0.620-0.852)	64.52% (46.88-78.95)	74.36% (58.76-85.59)	70.00% (58.41-79.51)
Radiomics model_AP	0.432	0.857 (0.800-0.913)	80.00% (69.06-87.82)	77.66% (68.18-84.97)	78.66% (71.74-84.27)	0.823 (0.728-0.918)	93.55% (78.25-99.24)	66.67% (50.91-79.44)	78.57% (67.50-86.67)
Radiomics model_PP	0.508	0.822 (0.756-0.887)	75.71% (64.42-84.33)	82.98% (74.02-89.35)	79.88% (73.05-85.34)	0.809 (0.709-0.909)	96.77% (82.42-100.82)	51.28% (36.20-66.14)	71.43% (59.89-80.73)
Radiomics model_HBP	0.471	0.831 (0.768-0.894)	71.43% (59.89-80.73)	84.04% (75.21-90.20)	78.66% (71.74-84.27)	0.809 (0.707-0.910)	74.19% (56.54-86.51)	74.36% (58.76-85.59)	74.29% (62.90-83.15)
Radiomics model_AP+PP+HBP	0.295	0.863 (0.809-0.918)	84.29% (73.84-91.17)	74.47% (64.76-82.25)	78.66% (71.74-84.27)	0.865 (0.783-0.947)	87.10% (70.54-95.48)	71.79% (56.10-83.58)	78.57% (67.50-86.67)
Integrated model	0.475	0.873 (0.821-0.925)	78.57% (67.50-86.67)	81.91% (72.84-88.49)	80.49% (73.71-85.87)	0.869 (0.789-0.950)	100.00% (86.91-100.00)	51.28% (36.20-66.14)	72.86% (61.39-81.95)

^*^Receiver operating characteristic analysis by maximising the Youden index.

AUC, area under the curve; PPV, positive predictive value; NPV, negative predictive value; LLR, lesion to liver ratio; AP, arterial phase; PP, portal phase; HBP, hepatobiliary phase; CI, confidence interval.

The calibration curves of the clinical-radiologic model, radiomics model, and integrated model for predicting VETC exhibited robust agreement across both the training and validation sets (**Figures 3C, D**). Decisive DCA curves were employed to predict VETC utilising the clinical-radiologic model, radiomics model, and integrated model, each showcased in **Figures 3E, F**. Two examples of the integrated model of the nomogram was shown in **Supplementary Figure 2**.

### 3.4 Predictors of survival

As of April 2022, 233 patients (99.57%) had concluded their recurrence-free survival (RFS) follow-up. The RFS rates at 1, 2, 3, 4, and 5 years stood at 93.8%, 86.4%, 78.9%, 75.1%, and 69.6%, respectively.

**Figure 4:**
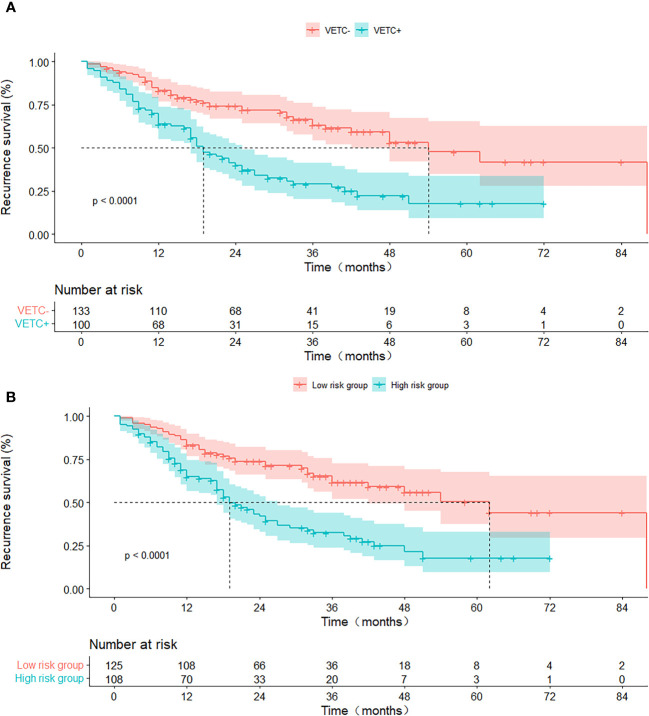
RFS curves stratified by histologic VETC pattern **(A)** and integrated model-predicted VETC pattern **(B)**, presented through Kaplan-Meier analysis. *VETC*, vessels encapsulating tumour clusters; *RFS*, recurrence-free survival.

In the entirety of the cohort (n = 233), VETC+ HCC demonstrated a notably inferior RFS when juxtaposed with VETC- HCC (all p < 0.05). The median RFS was 21 months (95% CI: 15.69–26.31) for those manifesting VETC and 54 months (95% CI: 43.09–80.91) for those without VETC (log-rank test, p < 0.001) (**Figure 4A**). Analogously, consistent findings emerged from the integrated model analysis: Patients predicted to possess the VETC pattern by the integrated model exhibited a median RFS of 21 months (95% CI: 13.32–25.51), whereas those predicted to lack VETC by the integrated model had a median RFS of 62 months (42.3–93.7) (log-rank test, p < 0.001) (**Figure 4B**).

## 4 Discussion

In this study, we systematically developed and validated clinico-radiologic, radiomics, and integrated models for preoperatively evaluating VETC in HCC. These models ingeniously incorporated pivotal clinical, radiological risk factors, and radiomics features extracted from gadoxetic acid-enhanced MRI. Our findings underscored the superior predictive capacity of the integrated model in comparison to the individual clinico-radiologic and radiomics models. Among the risk factors analysed, namely radiomics-AP, radiomics-HBP, peripheral washout, and necrosis or severe ischemia emerged as significant predictors.

In the validation set, the AUC (95% CI) of the integrated model was 0.869(0.789-0.950), the sensitivity was 100.00%(86.91-100.00), and the specificity was 71.28%(36.20-76.14). Recently, Dong et al (23) compares deep neural network and machine learning (ML) classifiers for evaluating VETC status, and finally a deep learning model performed well in the preoperative assessment of VETC status, the AUC was 0.844 (95% CI, 0.735–0.921), the sensitivity was 77.3%, and the specificity was 82.2%. However, in our study the AUC values, sensitivity, were slightly higher than theirs. Fan et al (16) used qualitative and quantitative imaging features of Gd-EOB-DTPA-enhanced MRI to investigate HCC with VETC, the AUC value of the nomogram was 0.885 (95% CI, 0.824–0.946). Their team (21) combined of the two texture features for identifying VETC-positive HCC achieved an AUC value of 0.844 (95% CI, 0.777, 0.910) with a sensitivity of 80.8% (95% CI, 70.1%, 91.5%) and specificity of 74.1% (95% CI, 64.5%, 83.6%). Yu et al (20) developed a Gd-EOB-DTPA-enhanced MRI radiomics model for preoperative prediction of VETC, the peritumoural radiomics model achieved an AUC of 0.909, however this study was only based on HBP and the qualitative feature analysis was not included in this study. Chen et al (17) developed the nomogram integrating gadoxetate disodium-enhanced MRI features for estimating VETC in HCC, showed good discrimination with a C-index of 0.870 (development cohort) and 0.862 (validation cohort), also in our study the AUC values were slightly higher than theirs. The study differed from previous works in that it analysed the imaging features of gadoxetic acid-enhanced MRI, based on the LI-RADS version 2018 criteria. As a standardised reporting system, LI-RADS v2018 is widely used in routine practice, and identifying imaging characteristics of relevant LI-RAD may help select appropriate treatment options and optimise the management of HCC patients. Therefore, this study tried to analyse the comprehensive image characteristics of the lesions as much as possible, and analysed the radiomics features from multiphase contrast-enhanced MRI.

Our findings highlighted a close relationship between the VETC pattern in HCC and specific attributes such as portal phase peripheral washout, necrosis or severe ischemia, as well as the presence of targetoid appearances during TP or HBP, along with the LLR_AP. The work of Feng et al. (9) demonstrated an independent connection between intratumour necrosis and the manifestation of the VETC pattern. As tumoural cells proliferate and expand, the resulting increased distance from the existing vascular supply induces hypoxia. Rapidly growing HCCs experience both new angiogenesis and hypoxia, leading to conspicuous necrosis (26). Notably, portal phase peripheral washout and targetoid appearances during the TP or HBP are likely to be associated with peripheral arterialisation and heightened cellular density, alongside central ischemia and necrosis. Therefore, while the presence of a targetoid appearance might suggest a non-HCC malignancy and trigger LI-RADS M categorisation, this does not preclude the possibility of HCC, particularly in cases of poorly differentiated HCC (27). The results of this study showed that the LLR arterial stage of VETC-positive HCC was lower than that of VETC-negative HCC, contrary to the study of Fan et al. (16). This discrepancy could potentially be attributed to the fact that VETC-positive HCC in our study exhibited a higher proportion of necrosis or severe ischemia.

Radiomics bears significant potential in enhancing clinical decision-making, especially in the realm of oncology (28). The VETC pattern encompasses heterogeneous angiogenesis within HCC behaviour, and in this study radiomic features were extracted from intratumoural regions, capturing biological properties linked to intratumoural heterogeneities. We conducted a rigorous canonical screening of radiomic features for the purpose of developing machine learning models, and subsequently employed validation sets to verify the stability of the integrated models. The AUC value (0.809-0.865) in the validation set of radiomics models were similar to the AUC value (0.822-0.873) in the training set, indicating that the model can be generalised from the training set to the validation set, and the performance is stable. For the model to gain broad acceptance and recognition, we will proceed to verify its robustness and generalisation capabilities in other cohorts in the future. Specifically, the radiomics model based on arterial phase (AP) data outperformed the radiomics models based on portal phase (PP) and hepatobiliary phase (HBP) data in the training and validation sets respectively. Our investigation encompassed 17 texture features, 4 first-order features, and a solitary original feature. These texture features, encompassing measures such as energy, homogeneity, correlation, entropy, dissimilarity, and second-order metrics (21), collectively captured intratumoural heterogeneity, aligning with previous research (20). Notably, the majority of features were derived from wavelet analysis, signifying that the features extracted from preprocessed images exhibited greater stability compared to those from the original images (20). Particularly, GLSZM features emerged at the forefront. Noteworthy examples include HBP_VOItumor_waveletHLL_glszm_SizeZoneNonUniformity and HBP_VOItumor_original_glszm_GrayLevelNonUniformity, which might relate to intratumoural texture heterogeneity attributed to factors like tumour cellularity, micronecrosis, and inflammation (23). In our study, wavelet analysis and GLSZM features demonstrated significant stability and predictive capability. Wavelet analysis captures different frequency components of the image through multi-scale decomposition, while GLSZM features reflect the spatial distribution of gray levels in the image. These features not only enhance model performance but also improve image resolution and feature extraction accuracy. The application of these features allows the model to better distinguish between different lesion types and demonstrates stronger capability in predicting patient prognosis. This finding partly elucidates the impressive performance of the VETC model. Intriguingly, 11 features were sourced from HBP data, highlighting the significance of gadoxetic acid-enhanced MRI in diagnosing VETC patterns.

Remarkably, the integrated model offered valuable information regarding the risk of VETC, which could potentially contribute to elucidating the correlation between tumour aggressiveness and prognosis. Moreover, the integrated model estimated VETC pattern had a similar result to the VETC status identified by CD34 immunostaining in predicting recurrence. The integrated model stratified patients into high-risk or low-risk VETC-positive HCCgroups. It may help clinicians define treatment plans, such as whether to consider the type of surgical resection, anatomic resection or non-anatomic resection, and whether to include adjuvant therapy (i.e., TACE). And sorafenib prolonged survival in patients with VETC-positive HCCs but not VETC-negative ones. Finally, given the observed responses of VETC-positive tumours to multikinase inhibitors (11), they may be considered before and after curative resection or liver transplant(ation) as (neo)adjuvant therapy to treat micrometastases and reduce recurrence. Thus, non-invasive identification of VETC-positive HCC can guide individualised management decisions, potentially selecting the treatment regimen that is likely to provide the greatest benefit to the individual patient.

Nonetheless, several limitations merit consideration in this study. Firstly, this was a single-center study, lack of external validation from other medical centres. Secondly, being retrospective, the study could potentially be influenced by selection bias. Thirdly, due to the short follow-up period for certain patients, the study excluded comprehensive overall survival analysis, potentially limiting its ability to comprehensively capture long-term outcomes and recurrence patterns. Lastly, our focus was exclusively on the VETC pattern; thus, exploring the interplay between the VETC pattern and other risk-related pathological factors, such as microvascular invasion (MVI), necessitates further investigation. Future studies will necessitate prospective studies and additional multi-center cohorts to further substantiate the integrated model. Furthermore, overall survival analysis will be include in these studies, thereby affording a deeper and more encompassing understanding of the prognostic significance of our integrated model. Future studies will explore whether the integration of MRI with other advanced imaging technologies (such as CT and PET) will further improve the accuracy and robustness of the predictive model. To enhance the interpretability of the model and mitigate the potential for overfitting, we aim to employ advanced image processing algorithms, while refining and optimising the radiomics feature extraction and selection procedure. Hepatitis C virus (HCV) is one of the leading causes of chronic liver disease, cirrhosis, and hepatocellular carcinoma, resulting in major global public health concerns (29). However, in this study the majority of patients were HBV-positive. The question of whether the integrated model is also applicable to HCV cohorts or patients with other liver diseases necessitates further investigation, so in the future an external sets with HCV will be included to prove the generalisation of the model. Indeed, the EMT pathway has been shown in mixed tumours with both VETC-positive and VETC-negative components, suggesting traditional HCC treatments are still required in addition to anti-VETC agents (30). Ultimately, we need to prospectively investigate interventions specific for VETC-positive (and VETC-negative) HCCs, including clinical treatment regimen or combination therapy regimen.

## 5 Conclusion

This study has developed an integrated model that combines traditional imaging features and radiomic features extracted from gadoxetic acid-enhanced MRI. The integrated model demonstrates good predictive performance for the VETC pattern in HCC.

## Data Availability

The raw data supporting the conclusions of this article will be made available by the authors, without undue reservation.
