# Supplementary material for: Radiomics analysis of gadoxetic acid-enhanced MRI for evaluating vessels encapsulating tumour clusters in hepatocellular carcinoma
Source: Front Oncol. 2024 Aug 13;14:1422119. doi: 10.3389/fonc.2024.1422119 (PMC11347320; doi:10.3389/fonc.2024.1422119)
Supplement: Supplementary file 1 [file DataSheet1.docx]

**Supplemental Materials**

**Supplementary Text 1.** Magnetic Resonance Imaging Examination

**Supplementary Text 2.** Quantitative image analysis

**Supplementary Table 1.** MRI scan sequence and parameters

**Supplementary Table 2.** Detailed Definitions of Imaging Features

**Supplementary Table 3.** Baseline Clinical Characteristics, Pathological Parameters, and Radiologic Factors of All Patients

**Supplementary Table 4.** Interobserver Agreement of Traditional Radiologic Features

**Supplementary Table 5.** Univariate and Multivariate Analysis of Preoperative Radiologic Factors in Predicting VETC

**Supplementary Table 6.** Formulae of the Radiomics Score

**Supplementary Fig 1.** Weighted Feature Coefficients of the Radiomics Model

**Supplementary Text 1. Magnetic Resonance Imaging Examination**

Magnetic Resonance Imaging (MRI) was executed using a 3.0 Tesla (T) whole-body MRI system (Intera Achieva 3.0 T, Philips Healthcare, Best, The Netherlands), equipped with a 16-channel phased array coil. All images were procured within the axial plane, following a fasting period of 4-6 hours. The administration of gadoxetic acid disodium (Gd-EOB-DTPA, Primovist; Bayer Schering, Pharma, Berlin, Germany) was employed as a contrast agent, with a dose of 0.025 mmol/kg body weight intravenously injected as a swift bolus via a power injector, at a rate of 2 ml/s, accompanied by a 20 ml NaCl flush. Preceding the introduction of the contrast agent, unenhanced MR images were obtained, encompassing fast spoiled dual-echo T1-weighted gradient-recalled-echo images (in-phase and out-phase), along with fat-suppressed fast spin-echo T2-weighted images (T2WI). Subsequent to the administration of Gd-EOB-DTPA, enhanced MR images were acquired in distinct phases: the arterial phase (20–35 s, AP), the portal phase (60 s, PP), the transitional phase (3 min, TP), and the hepatobiliary phase (20 min, HBP).

**Supplementary Text 2. Quantitative image analysis**

The tumour lesion-to-liver ratio (LLR) was ascertained through the measurement of tumour signal intensity before (SI pre) and after the intravenous introduction of Gd-EOB-DTPA during the arterial phase (AP), portal phase (PP), transitional phase (TP), and hepatobiliary phase (HBP), denoted as SI post. Regions of interest (ROI) were meticulously delineated through manual tracing, encompassing the maximal extent within the tumour region. Additionally, a distinct ROI was positioned within the liver parenchyma region, at a distance from vessels and excluding any focal hepatic lesions. The location of the ROI was deliberately maintained relatively consistent across each patient. The formula employed for computing the tumour LLR was: LLR = SI_tumour_ / SI_liver_.

**Supplementary Table 1. MRI scan sequence and parameters**

| Sequence | Orientation | Category | TR  (ms) | TE  (ms) | FOV  (mm) | Matrix | FA | Band  width(HZ) | Seam thickness (mm) | Fat  saturation | Hold breath |
| --- | --- | --- | --- | --- | --- | --- | --- | --- | --- | --- | --- |
| T2WI | TRA/ COR | TSE | 2000 | 70 | 325× 392 | 232× 202 | 90° | 513 | 7 | Yes | No |
| T1WI IN/OUT PHASE | TRA | T1-FFE | 150 | 2.3/1.15 | 375× 319 | 236× 200 | 55° | 1235 | 7 | Yes/No | Yes |
| T1WI+C | TRA/ COR | THRIVE | 3 | 1.5 | 385× 332 | 248×212 | 10° | 723.7 | 2.5 | Yes | Yes |

**Supplementary Table 2. Detailed Definitions of Imaging Features**

| LI-RADS major features | defination |
| --- | --- |
| Nonrim arterial phase hyperenhancement | Nonrim-like enhancement in arterial phase unequivocally greater in whole or in part than liver. |
| Nonperipheral washout | Nonperipheral visually assessed temporal reduction in enhancement of the liver observation in whole or in part relative to composite liver tissue in the portal venous phase |
| Enhancing capsule | Smooth, uniform, sharp border around most or all of a liver observation, unequivocally thicker or more conspicuous than fibrotic tissue around background nodules, and visible as enhancing rim in portal venous phase or transitional phase. |
| LI-RADS ancillary features（favoring HCC in particular） |  |
| Nonenhancing capsule | Capsule appearance not visible as an enhancing rim. |
| Mosaic architecture | Presence of randomly distributed internal nodules or compartments, usually with different imaging features |
| Nodule-in-nodule architecture | Nodule-in-nodule architecture: Presence of smaller inner nodule within and having different imaging features than larger outer nodule |
| Fat in mass | Fat in mass, more than adjacent liver:Excess fat within a mass, in whole or in part, relative to adjacent liver. |
| Blood products in mass | Intralesional or perilesional hemorrhage in the absence of biopsy, trauma or intervention |
| LI-RADS ancillary features (favoring malignancy, not HCC in particular) |  |
| Transitional phase hypointensity | Signal intensity of the liver observation in the transitional phase unequivocally less, in whole or in part, than liver. |
| Restricted diffusion | Signal intensity of the liver observation on diffusion-weighted imaging, not attributable solely to T2 shine-through, unequivocally higher than liver and/or apparent diffusion coefficient unequivocally lower than liver. |
| Mild-moderate T2 hyperintensity | Signal intensity of the liver observation on T2-weighted imaging mildly or moderately higher than liver and similar to or less than non-iron-overloaded spleen. |
| Corona enhancement | Periobservational enhancement in late arterial phase or early portal venous phase attributable to venous drainage from tumor. |
| Fat sparing in solid mass | Relative paucity of fat in solid mass relative to steatotic liver OR in inner nodule relative to steatotic outer nodule. |
| Hepatobiliary phase hypointensity | Signal intensity of the liver observation in the hepatobiliary phase unequivocally less, in whole or in part, than liver. |
| Iron sparing in solid mass | Paucity of iron in solid mass relative to iron-overloaded liver OR in inner nodule relative to siderotic outer nodule. |
| Rim arterial phase hyperenhancement | Rim-like enhancement of the liver observation in arterial phase unequivocally greater in whole or in part than liver. |
| LI-RADS M |  |
| Portal phase peripheral washout | Peripheral visually assessed temporal reduction in enhancement of the liver observation in whole or in part relative to composite liver tissue in the portal venous phase. |
| Delayed central enhancement | Central area of progressive postarterial phase enhancement. |
| Targetoid restriction | Concentric pattern on DWI characterized by restricted diffusion in observation periphery with relatively less restricted diffusion in observation center |
| Targetoid TP or HBP appearance | Concentric pattern in TP or HBP characterized by moderate-to-marked hypointensity in observation periphery with lesser degree of central hypointensity compared to background liver. |
| Infiltrative appearance | Non-circumscribed margin (indistinct transition) thought to represent malignancy with permeative growth. |
| Necrosis or severe ischemia | Area within a solid mass which either does not enhance at all (necrosis) or enhances very slowly and mildly (ischemia), not attributable to prior treatment. Suggests a poorly differentiated neoplasm that has “outgrown”its blood supply. |
| Non LI-RADS high-risk features |  |
| Non-Smooth tumor margin | Defined as a lobular or irregular protrusion into the surrounding normal hepatic parenchyma in any imaging plane. |
| Peritumoral hypointensity on HBP | Presence of wedge-shaped or flame-like hypointense area adjacent to the tumor border on hepatobiliary phase images. |

**References:**

American College of Radiology, CT/MRI Liver Imaging Reporting and Data System v2018 Core, American College of Radiology, America, 2018. https://www.acr.org/Clinical-Resources/Reporting-and-Data-Systems/LI-RADS/CT-MRI-LI-RADS-v2018.

**Supplementary Table 3. Baseline Clinical Characteristics, Pathological Parameters, and Radiologic Factors of All Patients**

| Characteristic | total (n=234) | VETC- (n=133) | VETC+ (n=101) | t/Z/x2 | P value * | training (n=163) | validation (n=71) | t/Z/x2 | P value * |
| --- | --- | --- | --- | --- | --- | --- | --- | --- | --- |
| Age (years) b | 58.00(52.00-65.00) | 60.00(53.00-66.00) | 57.00(51.00-63.00) | -2.614 | 0.009 | 59.00(52.00-66.00) | 57.00(52.00-64.00) | -0.943 | 0.346 |
| Sex (male) | 166(70.94) | 93(69.92) | 73(72.27) | 0.154 | 0.695 | 110(67.48) | 56(78.87) | 3.112 | 0.078 |
| AFP b | 11.20(3.45-122.08) | 8.38(3.77-70.03) | 17.95(3.28-206.87) | 1.113 | 0.266 | 11.51(3.28-122.83) | 10.80(3.99-101.10) | 0.109 | 0.913 |
| PIVKA-II b | 69.17(30.15-314.27) | 44.36(21.58-180.38) | 142.49(58.84-384.36) | 4.086 | <0.001 | 91.48(25.63-323.49) | 68.33(30.15-314.27) | -0.473 | 0.637 |
| CEA b | 2.41(1.59-3.73) | 2.46(1.66-3.85) | 2.27(1.48-3.45) | -1.038 | 0.299 | 2.33(1.39-3.56) | 2.58(1.81-3.83) | 1.065 | 0.287 |
| CA199 b | 16.84(8.99-28.65) | 17.65(9.01-31.26) | 15.51(8.99-27.56) | -0.687 | 0.492 | 16.33(8.95-28.33) | 17.81(9.01-31.72) | 0.426 | 0.670 |
| INR b | 1.03(0.96-1.11) | 1.02(0.95-1.13) | 1.03(0.97-1.09) | 0.755 | 0.450 | 1.03(0.96-1.12) | 1.03(0.95-1.11) | -0.141 | 0.888 |
| ALB a | 40.58±4.96 | 40.70±5.32 | 40.42±4.46 | 0.440 | 0.662 | 40.21±4.71 | 41.42±5.42 | -1.710 | 0.088 |
| TBIL b | 16.30(11.90-21.00) | 16.50(12.70-21.00) | 15.90(11.70-20.80) | -0.444 | 0.657 | 16.00(11.50-20.90) | 16.50(12.70-21.30) | 0.985 | 0.325 |
| Scr b | 66.95(56.10-75.00) | 66.00(56.10-75.00) | 67.00(56.40-74.20) | 0.271 | 0.786 | 66.00(56.00-75.00) | 68.00(61.00-77.20) | 0.953 | 0.341 |
| ALT b | 31.50(21.00-52.00) | 30.00(22.00-51.00) | 36.00(20.00-52.00) | 0.106 | 0.915 | 32.00(21.00-53.00) | 30.00(22.00-48.00) | 0.025 | 0.980 |
| AST b | 37.00(25.00-53.00) | 37.00(26.00-53.00) | 37.00(25.00-51.00) | -0.232 | 0.817 | 37.00(25.00-54.00) | 37.00(27.00-49.00) | 0.340 | 0.734 |
| ALP b | 87.00(66.00-112.00) | 87.00(67.00-113.00) | 89.00(62.00-110.00) | -0.640 | 0.523 | 87.00(62.00-109.00) | 89.00(68.00-122.00) | 0.982 | 0.326 |
| GGT b | 44.50(27.00-79.00) | 44.00(27.00-80.00) | 45.00(30.00-75.00) | 0.365 | 0.715 | 43.00(27.00-71.00) | 49.00(25.00-96.00) | 1.314 | 0.189 |
| PLT b | 113.50(77.00-157.00) | 115.00(77.00-157.00) | 112.00(79.00-164.00) | -0.004 | 0.997 | 115.00(78.00-171.00) | 112.00(72.00-146.00) | -0.858 | 0.391 |
| PT b | 11.90(11.10-12.90) | 11.90(11.00-13.10) | 12.00(11.30-12.70) | 0.696 | 0.486 | 11.90(11.20-12.90) | 11.90(11.10-12.90) | -0.163 | 0.871 |
| APRI b | 0.34(0.20-0.64) | 0.38(0.19-0.69) | 0.31(0.22-0.54) | -0.356 | 0.722 | 0.32(0.20-0.60) | 0.37(0.20-0.66) | 0.712 | 0.476 |
| Cause of liver disease(HBV,others) | 211,23 | 120,13 | 91,10 |  | 0.901 | 150(92.02) | 61(85.92) |  | 0.161 |
| Child-Pugh(A,B) | 200,34 | 117,16 | 83,18 | 1.551 | 0.213 | 142,21 | 58,13 | 1.173 | 0.279 |
| Diameter（cm）b | 2.80(1.70, 5.10) | 2.60 （1.50, 4.00） | 3.00 （2.00, 4.60） | 2.675 | 0.016 | 2.60(1.70,4.20) | 2.70(1.80, 4.20) | 1.986 | 0.005 |
| Number of tumor(sigle,multi) | 192,42 | 115,18 | 77,24 | 4.078 | 0.043 | 137,26 | 55,16 | 1.456 | 0.228 |
| MVI(%) | 63(26.92) | 18(13.53) | 45(44.55) | 28.077 | <0.001 | 40(24.53) | 23(32.39) | 1.551 | 0.213 |
| Edmondson grade（I-II,III-IV） | 46,188 | 39,94 | 7,94 | 18.226 | <0.001 | 36,127 | 10,61 | 2.005 | 0.157 |
| MTM(%) | 12(5.13) | 0 | 12(11.88) | 16.656 | <0.001 | 10(6.13) | 2(2.82) |  | 0.355 |
| Capsule infiltration | 21(8.97) | 7(5.26) | 14(13.86) | 5.195 | 0.023 | 13(7.98) | 8(11.27) | 0.656 | 0.418 |
| Inflammatory infiltrates | 169(72.22) | 106(79.70) | 63(62.38) | 8.587 | 0.003 | 119(73.01) | 50(70.42) | 0.165 | 0.685 |
| Gross vascular invasion | 9(3.85) | 4(3.01) | 5(4.95) |  | 0.505 | 6(3.68) | 3(4.22) |  | 1.000 |
| Satellite nodule | 9(3.85) | 3(2.26) | 6(5.94) |  | 0.180 | 3(1.84) | 6(8.45) |  | 0.024 |
| CK7+(%) | 126(53.85) | 78(58.65) | 48(47.52) | 2.857 | 0.091 | 83(50.92) | 43(60.56) | 1.851 | 0.174 |
| CK19+(%) | 60(25.64) | 37(27.82) | 23(22.77) | 0.767 | 0.381 | 40(24.54) | 20(28.17) | 0.342 | 0.559 |
| Radiology Factors |  |  |  |  |  |  |  |  |  |
| Nonrim arterial phase hyperenhancement（%） | 133(56.84) | 83(62.41) | 50(37.59) | 3.895 | 0.048 | 96(58.90) | 37(52.11) | 0.928 | 0.336 |
| Nonperipheral washout（%） | 134(57.26) | 84(63.16) | 50(37.31) | 4.373 | 0.037 | 97(59.51) | 37(52.11) | 1.106 | 0.293 |
| Enhancing capsule（%） | 175(74.79) | 93(69.92) | 82(46.86) | 3.862 | 0.049 | 113(69.33) | 62(87.32) | 8.497 | 0.004 |
| Nonenhancing capsule（%） | 10(4.27) | 3(2.26) | 7(70.00) |  | 0.105 | 8(4.91) | 2(2.82) |  | 0.727 |
| Mosaic architecture（%） | 23(9.83) | 15(11.28) | 8(34.78) | 0.730 | 0.393 | 16(9.82) | 7(9.86) | 0.000 | 0.992 |
| Nodule-in-nodule architecture（%） | 11(4.70) | 10(7.52) | 1(9.09) |  | 0.026 | 8(4.91) | 3(4.22) |  | 1.000 |
| Fat in mass（%） | 44(18.80) | 22(16.54) | 22(50.00) | 1.033 | 0.310 | 30(18.40) | 14(19.72) | 0.056 | 0.813 |
| Blood products in mass（%） | 38(16.24) | 20(15.04) | 18(47.37) | 0.327 | 0.567 | 24(14.72) | 14(19.72) | 0.907 | 0.341 |
| Transitional phase hypointensity（%） | 232(99.15) | 132(99.25) | 100(43.10) |  | 1.000 | 161(98.77) | 71(100) |  | 1.000 |
| Restricted diffusion（%） | 233(99.57) | 132(99.25) | 101(43.35) |  | 1.000 | 162(99.39) | 71(100) |  | 1.000 |
| Mild-moderate T2 hyperintensity（%） | 233(99.57) | 132(99.25) | 101(43.35) |  | 1.000 | 162(99.39) | 71(100) |  | 1.000 |
| Corona enhancement（%） | 73(31.20) | 39(29.32) | 34(46.58) | 0.504 | 0.478 | 48(29.45) | 25(35.21) | 0.765 | 0.382 |
| Fat sparing in solid mass（%） | 186(79.49) | 110(82.71) | 76(40.86) | 1.959 | 0.162 | 129(79.14) | 57(80.28) | 0.040 | 0.843 |
| Hepatobiliary phase hypointensity（%） | 233(99.57) | 132(99.25) | 101(43.35) |  | 1.000 | 163(100) | 70(98.59) |  | 0.303 |
| Iron sparing in solid mass（%） | 228(97.44) | 129(96.99) | 99(43.42) |  | 0.701 | 158(96.93) | 70(98.59) |  | 0.671 |
| Rim arterial phase hyperenhancement（%） | 94(40.17) | 45(33.83) | 49(52.13) | 5.148 | 0.023 | 63(38.65) | 31(43.66) | 0.517 | 0.472 |
| Portal phase peripheral washout（%） | 59(25.21) | 20(15.04) | 39(66.10) | 16.922 | <0.001 | 40(24.54) | 19(26.76) | 0.129 | 0.719 |
| Delayed central enhancement（%） | 8(3.42) | 6(4.51) | 2(25.00) |  | 0.471 | 8(4.91) | 0(0.00) |  | 0.110 |
| Targetoid restriction（%） | 64(27.35) | 34(25.56) | 30(46.88) | 0.495 | 0.482 | 42(25.77) | 22(30.99) | 0.678 | 0.410 |
| Targetoid TP or HBP appearance（%） | 52(22.22) | 32(24.06) | 20(38.46) | 0.602 | 0.438 | 42(25.77) | 22(30.99) | 0.678 | 0.410 |
| Infiltrative appearance （%） | 37(15.81) | 20(15.04) | 17(45.95) | 0.139 | 0.710 | 27(16.56) | 10(14.08) | 0.229 | 0.633 |
| Necrosis or severe ischemia（%） | 66(28.21) | 22(16.54) | 44(66.67) | 20.702 | <0.001 | 46(28.22) | 20(28.17) | 0.000 | 0.994 |
| Non-Smooth tumor margin（%） | 61(26.07) | 32(24.06) | 29(47.54) | 0.645 | 0.422 | 42(25.77) | 19(26.76) | 0.025 | 0.874 |
| Peritumoral hypointensity on HBP（%） | 83(35.47) | 43(32.33) | 40(48.19) | 1.327 | 0.249 | 51(31.29) | 32(45.07) | 4.104 | 0.043 |
| LLR_AP b | 1.28(1.21-1.50) | 1.40(1.22-1.66) | 1.24(1.21-1.31) | -4.562 | <0.001 | 1.30(1.22-1.44) | 1.24(1.18-1.56) | -0.783 | 0.434 |
| LLR_PP b | 0.54(0.46-0.62) | 0.55(0.46-0.62) | 0.52(0.46-0.61) | -1.494 | 0.135 | 0.54(0.46-0.62) | 0.54(0.46-0.61) | 0.122 | 0.903 |
| LLR_HBP b | 0.94(0.86-1.08) | 0.98(0.87-1.07) | 0.87(0.72-0.98) | -3.545 | <0.001 | 0.89(0.79-0.99) | 0.87(0.82-1.02) | -0.608 | 0.564 |

Notes: Categorical variables are presented as N (%) according to different levels.

^a^ Data are presented as mean ± standard deviation

^b^ Data are presented as median (interquartile range)

Abbreviations: *AFP*, alpha-fetoprotein; *PIVKA-II,* protein induced by vitamin K absence-II; *CEA*,carcinoembryonic antigen;*CA199*, carbohydrate antigen 199; *ALT*, alanine aminotransferase; *AST*, aspartate aminotransferase; *ALB,* serum albumin; *TB*, serum total bilirubin ; *GGT*, gamma-glutamyl transferase; *ALP*, alkaline phosphatase; *PLT,* platelet count; *PT*, prothrombin time; *INR*, international normalized ratio; *Scr*, serum creatinine ; APRI=AST/PLT; *HBV*, hepatitis B virus;*MVI*, Microvascular invasion; *MTM*, Macrotrabecular-massive; *LI-RADS*, Liver Imaging Reporting and Data System; *LLR*, Lesion to liver ratio; *AP*, arterial phase; *PP*, portal phase; *HBP*, hepatobiliary phase; *TP*, transitional phase

^*^ *T* test results for continuous variables with normal distribution, Wilcoxon test results for continuous variables with abnormal distribution, and chisquare test or Fisher exact test results for categorical variables

**Supplementary Table 4. Interobserver agreement of traditional radiologic features**

| *LI-RADS major features* | Kappa value |
| --- | --- |
| Nonrim arterial phase hyperenhancement | 0.879 |
| Nonperipheral washout | 0.806 |
| Enhancing capsule | 0.763 |
| *LI-RADS ancillary features（favoring HCC in particular）* |  |
| Nonenhancing capsule | 0.702 |
| Mosaic architecture | 0.896 |
| Nodule-in-nodule architecture | 0.781 |
| Fat in mass | 0.963 |
| Blood products in mass | 0.954 |
| *LI-RADS ancillary features (favoring malignancy, not HCC in particular)* |  |
| Transitional phase hypointensity | 1 |
| Restricted diffusion | 1 |
| Mild-moderate T2 hyperintensity | 1 |
| Corona enhancement | 0.764 |
| Fat sparing in solid mass | 0.901 |
| Hepatobiliary phase hypointensity | 1 |
| Iron sparing in solid mass | 0.734 |
| Rim arterial phase hyperenhancement | 0.701 |
| *LI-RADS M features* |  |
| Portal phase peripheral washout | 0.703 |
| Delayed central enhancement | 0.631 |
| Targetoid restriction | 0.793 |
| Targetoid TP or HBP appearance | 0.924 |
| Infiltrative appearance | 0.902 |
| Necrosis or severe ischemia | 0.978 |
| *Non LI-RADS high-risk features* |  |
| Non-Smooth tumor margin | 0.972 |
| Peritumoral hypointensity on HBP | 0.989 |
| *quantitative indicators* |  |
| LLR_AP | 0.953 |
| LLR_PP | 0.752 |
| LLR_HBP | 0.978 |

Abbreviations: *LI-RADS*, Liver Imaging Reporting and Data System; *LLR*, Lesion to liver ratio; *AP*, arterial phase; *PP*, portal phase; *HBP*, hepatobiliary phase; *TP*, transitional phase

**Supplementary Table 5. Univariate and Multivariate Analysis of Preoperative Radiologic Factors in Predicting VETC**

| Factors | Univariate analysis | | | Multivariate analysis | | |
| --- | --- | --- | --- | --- | --- | --- |
|  | OR | 95 % CI | p value | OR | 95 % CI | p value |
| Nonperipheral washout | 0.523 | 0.277-0.986 | 0.045 |  |  |  |
| Enhancing capsule | 2.015 | 1.001-4.055 | 0.049 |  |  |  |
| Rim arterial phase hyperenhancement | 2.222 | 1.168-4.228 | 0.015 |  |  |  |
| Portal phase peripheral washout | 6 | 2.675-13.459 | <0.001 | 6.493 | 2.485-16.967 | <0.001 |
| Necrosis or severe ischemia | 3.978 | 1.926-8.215 | <0.001 | 4.756 | 1.964-11.516 | <0.001 |
| Targetoid TP or HBP appearance | 0.884 | 0.411-1.903 | 0.043 | 1.307 | 0.101-1.935 | 0.038 |
| LLR_AP | 0.082 | 0.017-0.385 | 0.002 | 0.082 | 0.012-0.459 | 0.005 |

Abbreviations: *VETC*, Vessels encapsulating tumor clusters; *LLR*, Lesion to liver ratio; *AP*, arterial phase; *PP*, portal phase; *HBP*, hepatobiliary phase; *TP*, transitional phase; *CI*, Confidence interval; *OR*,Odds ratio

**Supplementary Table 6. Formulae of the radiomics score**

| Radiomics score | Calculation formula |
| --- | --- |
| Radiomics -AP | 0.6481506615964778 ＋  0.0592512049358645×AP_original_firstorder_Entropy  -0.1094819869231512 × AP_wavelet-LLH_glrlm_ShortRunLowGrayLevelEmphasis  -0.08789525261379873×AP_wavelet-LLH_glcm_SumAverage  ＋3.769230769230769 × AP_wavelet-LHH_glcm_ClusterShade  -0.2222222222222222 × AP_wavelet-LHH_glszm_SizeZoneNonUniformity  -0.17671556243493802 ×AP_wavelet-LHH_glszm_SizeZoneNonUniformityNormalized  -0.3819769758216203 × AP_wavelet-LHH_glszm_SmallAreaEmphasis＋0.05141224663294774×AP_wavelet-LLH_gldm_DependenceEntropy  -0.12862876456535083 × AP_wavelet-HLH_firstorder_Mean  -0.7400863552012511× AP_wavelet-HHL_glcm_ClusterShade  ＋ 0.04826460724701469 × AP_wavelet-HHL_glszm_GrayLevelNonUniformity |
| Radiomics -PP | 0.00455461248285322  ＋0.00151195273205304×PP_original_shape_MajorAxisLength  -0.00123909541538783 × PP_diagnostics_Image-original_Mean  ＋0.00125178165723594×PP_original_glcm_Correlation  -0.025415088201662917 × PP_wavelet-LHH_firstorder_Mean  ＋ 0.000342801568930041 × PP_wavelet-HHH_gldm_LargeDependenceHighGrayLevelEmphasis |
| Radiomics -HBP | 0.51181839335065627  -0.37811794761936324 × HBP_original_glszm_SizeZoneNonUniformityNormalized  ＋ 0.3125015670531639 × HBP_original_shape_LeastAxisLength  ＋ 5.061728395061729 × HBP_original_glszm_ZoneVariance  ＋ 0.034689307752619185 × HBP_log-sigma-4-0-mm-3D_glcm_ClusterShade  ＋ 5.823529411764706 × HBP_log-sigma-4-0-mm-3D_gldm_LargeDependenceHighGrayLevelEmphasis  --0.002757060250105807 × HBP_wavelet-LLH_glszm_SmallAreaLowGrayLevelEmphasis  ＋ 0.023513160607174576 × HBP_wavelet-LHH_firstorder_Minimum  ＋ 0.017692801276508165 × HBP_wavelet-LHH_firstorder_Skewness  -4.397382336050656e-09 × HBP_wavelet-LHH_glszm_ZoneVariance  -0.2231041438901418 × HBP_wavelet-HLL_glrlm_ShortRunLowGrayLevelEmphasis  -0.22322081243106318× HBP_wavelet-HLH_glszm_SmallAreaEmphasis  ＋ 0.10394943263660802 × HBP_wavelet-LLL_glszm_GrayLevelNonUniformity |
| Radiomics-AP+PP+HBP | 0.6971819496198046 ＋  0.24211669181509785 × AP_wavelet-LLH_glrlm_ShortRunLowGrayLevelEmphasis  -0.0994619784604328 × AP_wavelet-LHH_glcm_ClusterShade  -0.06311348182738522 × AP_wavelet-LHH_glszm_SizeZoneNonUniformity  -0.17671556243493802 ×AP_wavelet-LHH_glszm_SizeZoneNonUniformityNormalized  -0.1455060367900937 × AP_wavelet-LHH_glszm_SmallAreaEmphasis  -0.012015721172605984 × AP_wavelet-HLH_firstorder_Mean  -0.7271674648212303 × AP_wavelet-HHL_glcm_ClusterShade  ＋ 0.015385080193530691 × AP_wavelet-HHL_glszm_GrayLevelNonUniformity  -0.25735769424960103 × HBP_original_glszm_SizeZoneNonUniformityNormalized  ＋ 1.4694809154362396e-07 × HBP_original_glszm_ZoneVariance  ＋ 0.09547464140096815 × HBP_log-sigma-4-0-mm-3D_glcm_ClusterShade  ＋ 4.3229548930785784e-05 × HBP_log-sigma-4-0-mm-3D_gldm_LargeDependenceHighGrayLevelEmphasis  -0.5045502022997875 × HBP_wavelet-LLH_glszm_SmallAreaLowGrayLevelEmphasis  ＋ 0.023513160607174576 × HBP_wavelet-LHH_firstorder_Minimum  ＋ 0.07957969084471067 × HBP_wavelet-LHH_firstorder_Skewness  -4.397382336050656e-09 × HBP_wavelet-LHH_glszm_ZoneVariance  ＋0.11594478241139002 × HBP_wavelet-HLL_glrlm_ShortRunLowGrayLevelEmphasis  -0.018557576609485548 × HBP_wavelet-HLH_glszm_SmallAreaEmphasis  ＋ 0.013821419585868149 × HBP_wavelet-LLL_glszm_GrayLevelNonUniformity  -0.018122253680829994 × PP_diagnostics_Image-original_Mean  -0.025415088201662917 × PP_wavelet-LHH_firstorder_Mean  ＋ 0.011679760727170105 × PP_wavelet-HHH_gldm_LargeDependenceHighGrayLevelEmphasis |

Abbreviations: *LI-RADS*, Liver Imaging Reporting and Data System; *LLR*, Lesion to liver ratio; *AP*, arterial phase; *PP*, portal phase; *HBP*, hepatobiliary phase


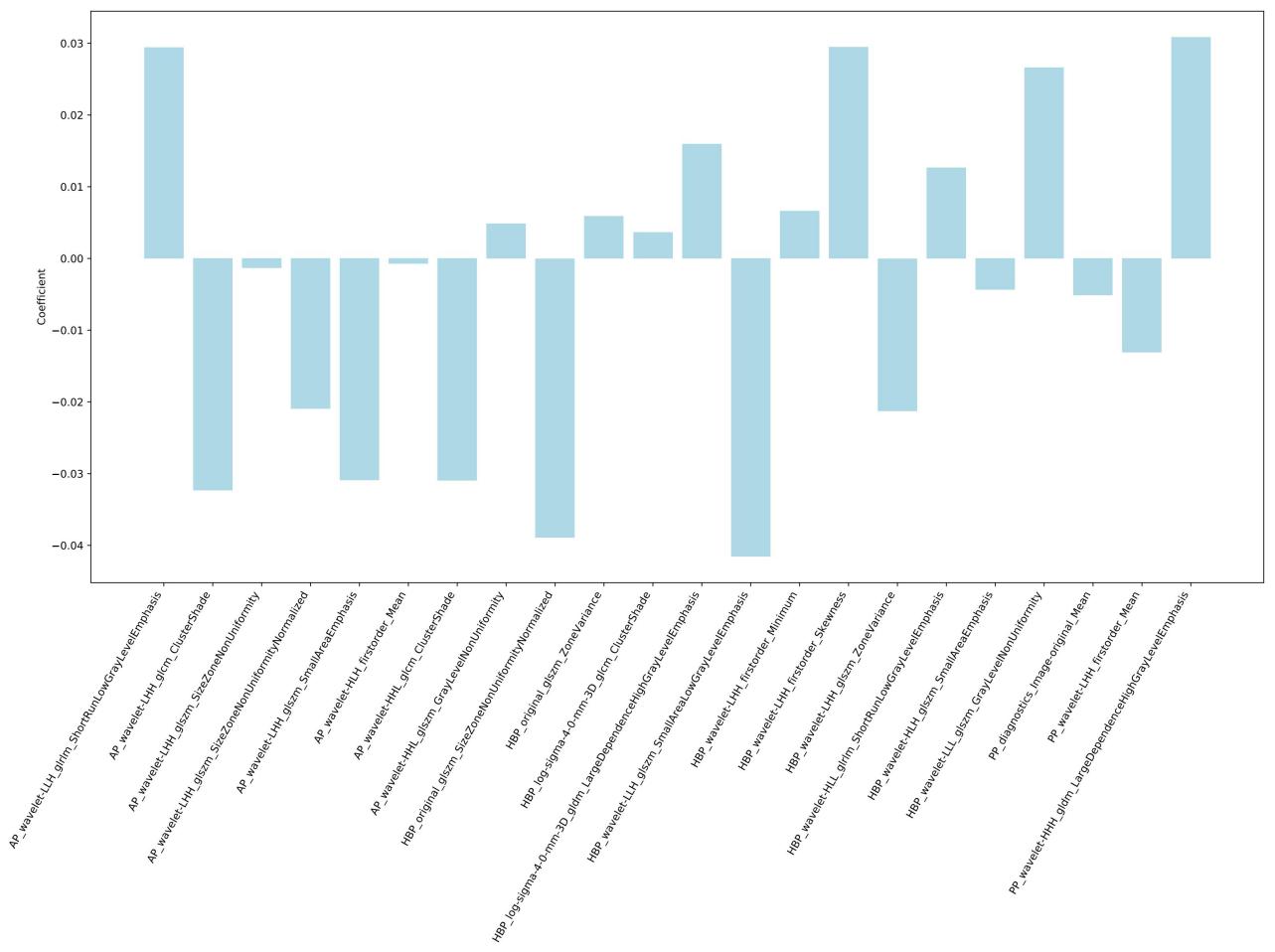


**Supplementary Fig 1.** **Weighted Feature Coefficients of the Radiomics Model**

j

e

i

h

g

f

d

c

b

a


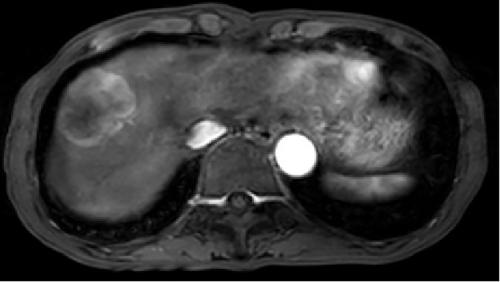

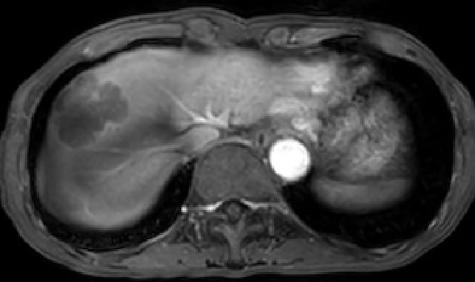

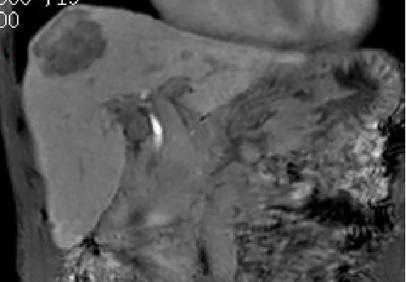

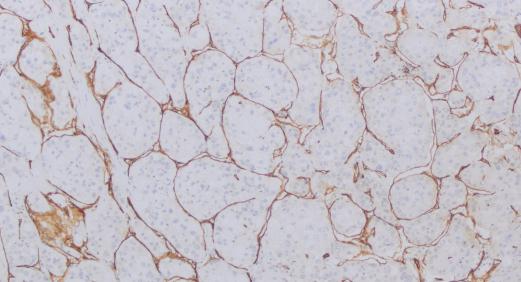

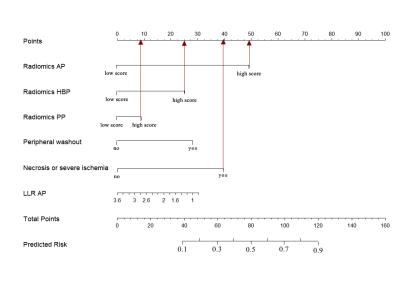

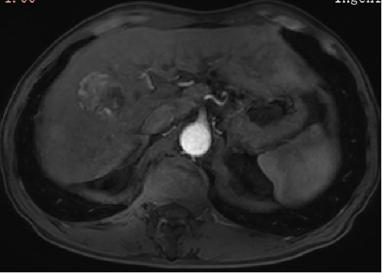

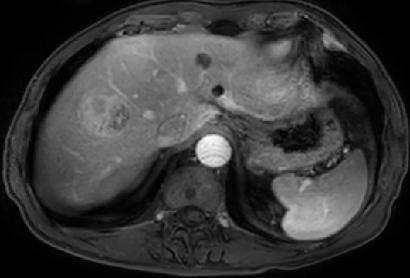

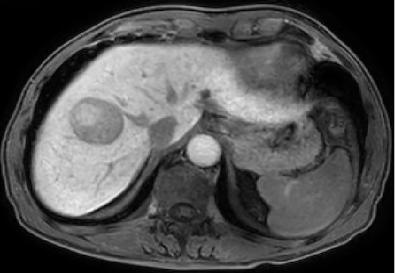

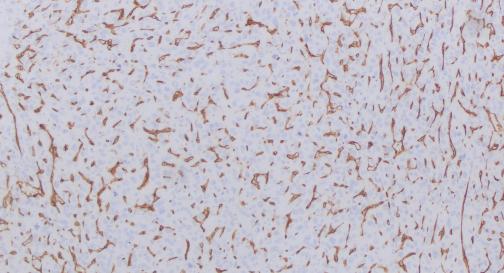

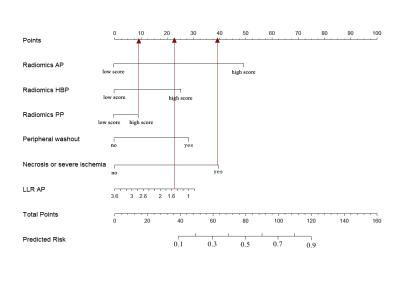


**Supplementary Fig 2.** Two examples of the integrated model of the nomogram.(a-e)These images

from a 71-year-old male patient with HCC in VETC-positive group. (a)AP image. (b)PP image.(c) HBP image.(d) Immunohistochemical staining for VETC positive pattern (original magnifications x 100). (e) The factors in the nomogram were analyzed as follows:

The value of radiomics AP, radiomics PP, radiomics HBP are greater than their cut-off values. Necrosis or severe ischemia= “yes”.The total score was 114 and the probability of VETC-positive was 0.84. (f-j) These images from a 67-year-old male patient with HCC in VETC-negative group. (f) AP image. (g) PP image.(h) HBP image.(i) Immunohistochemical staining for VETC positive pattern (original magnifications x 100). (j) The factors in the nomogram were analyzed as follows: Only the value of radiomics PP is greater than their cut-off values. Necrosis or severe ischemia= “yes”.LLR_AP=1.5. The total score was 70 and the probability of VETC-positive was 0.4.

Abbreviations: *HCC,* hepatocellular carcinoma; *VETC*, vessels encapsulating tumor clusters; *AP*, arterial phase; *PP*, portal phase; *HBP*, hepatobiliary phase
